# [^18^F]FDG PET/CT performs better than CT in determining the bone biopsy site : randomized controlled clinical trial

**DOI:** 10.1186/s40644-024-00804-6

**Published:** 2024-11-24

**Authors:** Yujie Chang, Yifeng Gu, Shunyi Ruan, Shengyuan Xu, Jing Sun, Zhiyuan Jiang, Guangyu Yao, Zhiyu Wang, Hui Zhao

**Affiliations:** 1 Internal Oncology, Shanghai Jiao Tong University Affiliated Sixth People’s Hospital, Shanghai, 200233 China; 2 Interventional Radiology, Shanghai Jiao Tong University Affiliated Sixth People’s Hospital, Shanghai, China; 3 Mailman School of Public Health, Columbia University, New York, USA

**Keywords:** Bone metastasis, Diagnostic test, Imaging, Positron emission tomography, Computed tomograph, Clinical trial results

## Abstract

**Background:**

Bone biopsy is the gold standard for diagnosing bone metastases. However, there is no clinical consensus regarding the optimal imaging test for determining the puncture site.

**Methods:**

We compared the performance of [^18^F]FDG PET/CT with CT in detecting bone metastases to achieve the highest biopsy efficiency. This registered prospective study enrolled 273 patients with bone lesions who were treated between January 2020 and March 2021. Patients were randomly assigned to undergo [^18^F]FDG PET/CT or CT to determine the puncture site before bone biopsy. The accuracy, sensitivity, specificity, second biopsy rate, diagnostic time and cost-effectiveness of the two imaging tests were compared.

**Results:**

The accuracy and sensitivity of [^18^F]FDG PET/CT group in detecting bone metastases were significantly higher than CT group(97.08% vs. 90.44%, 98.76% vs. 92.22%, *P *< 0.05). The second biopsy rate was significantly lower in the [^18^F]FDG PET/CT group (2.19% vs. 5.15%; *P* < 0.05). The diagnostic time of [^18^F]FDG PET/CT was 18.33 ± 2.08 days, which was significantly shorter than 21.28 ± 1.25 days in CT group ( *P *< 0.05). The cost of [^18^F] FDG PETCT is 11428.35 yuan, and the cost of CT is 13287.52 yuan; the incremental cost is 1859.17 yuan. SUVmax > 6.3 combined with ALP > 103 U/L showed a tendency for tumor metastases with an AUC of 0.901 (95%CI 0.839 to 0.946, *P *< 0.001).

**Conclusion:**

[^18^F]FDG PET/CT has better performance and cost-effectiveness than CT in determining the bone biopsy site for suspect bone metastases.

**Trial registration:**

The prospective study was registered on 2018-04-10, and the registration number is ChiCTR1800015540.

**Supplementary Information:**

The online version contains supplementary material available at 10.1186/s40644-024-00804-6.

## Background

Bone is a common site of metastatic solid tumors [1]. More than 50% of all cancers develop bone metastases [2]. Bone is the third most common site of tumor metastasis, with only the lungs and liver having a higher metastatic rate [3]. Bone metastases often cause skeletal complications known as skeletal-related events (SREs), including pathological fractures, radiotherapy, bone surgery, spinal cord compression, and hypercalcemia. SREs can cause loss of mobility and social functioning, further reducing the quality of life (QoL), increasing healthcare expenditure, and worsening survival [4]. Early evaluation and diagnosis of bone metastases are important to ensure effective treatment.

Pathological examination, which mainly involves bone biopsy, is the gold standard for the diagnosis of malignant bone metastasis. Biopsy of bone lesions has a diagnostic accuracy ranging from 66 to 98%, which is a significant difference [5]. Complications of bone biopsy include pain, osteomyelitis, and hematoma. If the metastatic site is adjacent to the lungs or spinal cord, puncture may cause pneumothorax and nerve root irritation [6, 7]. Owing to the risks of invasive examinations, conducting imaging tests before biopsies to determine the puncture sites can improve the success rate of diagnosis and minimize potential complications.

Imaging tests, including bone scintigraphy, computed tomography (CT) and [^18^F] fluorodeoxyglucose positron emission tomography/computed tomography ([^18^F]FDG PET/CT) are commonly used for screening bone metastases. Bone scintigraphy is highly sensitive but usually has low specificity [8]. The sensitivity and specificity of CT in detecting malignant bone metastases are not superior to those of other traditional imaging tools (including bone scintigraphy) [9]. [^18^F]FDG PET/CT is a promising tool that combines metabolic index standardized uptake values (SUV) with traditional imaging tools. Metabolically active bone lesions on [^18^F]FDG PET/CT can result from primary or metastatic malignant tumors or benign bone diseases [10]. SUVmax has been proven to be a metabolic parameter in oncology [11]. It can be used as a reliable semi-quantitative indicator to differentiate metastatic bone lesions from normal tissues. Although [^18^F]FDG PET/CT has several clinical advantages, it is not covered by basic medical insurance in China; therefore, undergoing [^18^F]FDG PET/CT can increase the financial burden of patients. Therefore, CT remains the primary choice for pre-biopsy imaging to determine optimal puncture sites. However, secondary punctures for CT localization may also increase diagnostic time and patient economic burden due to the poor accuracy of CT-localized biopsies.

Malignant tumor metastasis mainly involves bone remodeling, including bone resorption and formation. Serum calcium, phosphorus, alkaline phosphatase (ALP), and other indicators can indicate bone turnover and evaluate the progression of bone lesion progress [1]. Serological examinations are widely used in clinical practice to inspect bone metastases because of the convenience of noninvasive detection [12]. These indicators must be complemented by imaging test [13].

Consequently, this prospective study aimed to determine whether [^18^F]FDG PET/CT could determine a puncture site more accurately than CT to improve the diagnostic rate of biopsy. Moreover, we attempted to determine the best cutoff value of clinical indicators for differentiating malignant bone metastases using a noninvasive examination and [^18^F]FDG PET/CT.

## Methods

### Study design and participants

This prospective, single-center, comparative imaging study was approved by the Ethics Committee of the Shanghai Sixth People’s Hospital and registered in the Chinese Clinical Trial Registry (ChiCTR1800015540). The principles of the Declaration of Helsinki were adhered to. Written informed consent was obtained from all patients.

The inclusion criteria were: (1) referral for diagnostic workup for bone disease, (2) age older than 18 years, and (3) Karnofsky performance status of at least 60.

The exclusion criteria were: (1) contraindications for biopsies (such as infection at the puncture sites), (2) severe bleeding metastases (such as severe hemophilia or severe disseminated intramuscular coagulopathy), and (3) random blood glucose > 11.1 mmol/L.

### Procedures

After being included according to the abpve criteria, all patients were randomly assigned to the CT or [^18^F]FDG PET/CT group at a ratio of 1:1. The two groups underwent the corresponding imaging tests separately to determine the puncture sites before diagnostic biopsies. The demographic characteristics, SUVmax of [^18^F]FDG PET/CT, and serological test results (including serum ALP, calcium, and phosphorus) of the two groups were collected from our center and analyzed using a de-identification method.

### Procedure of [18F]FDG PET/CT and CT

**Fig. 1:**
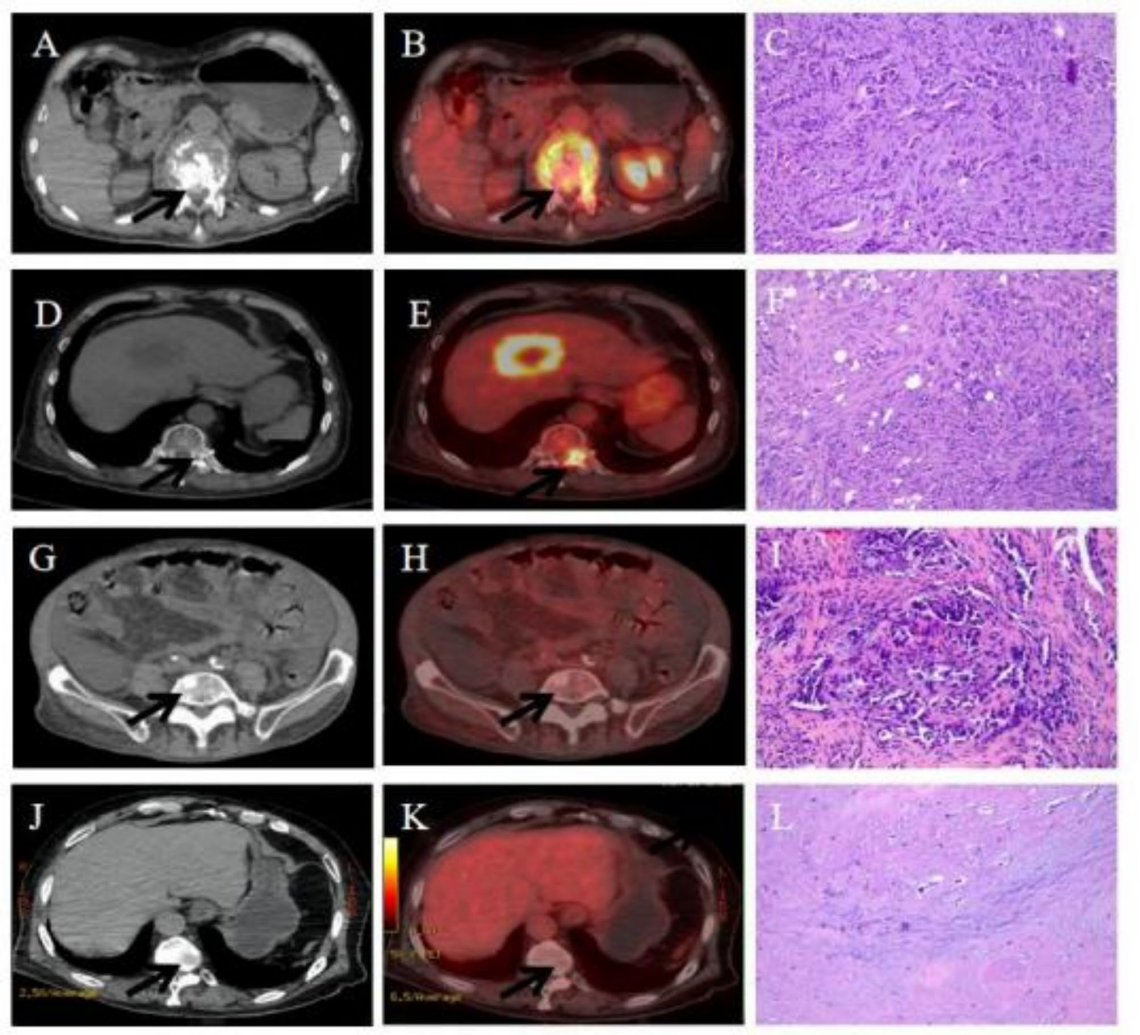
[^18^F]FDG PET/CT, CT and bone biospy images of bone metastases are shown. A 63-year-old man who represented with lytic metastatic lesions of spine with lung adenocarcinoma (**A**-**C**), the possible bone metastasis showed in CT and [^18^F]FDG PET/CT. The HE staining confirmed tumor metastasis. A 67-year-old man with no abnormal lesions was seen in CT (**D**), while a hypermetabolic bone lesion was showed in [^18^F]FDG PET/CT (**E**). Bone biospy of hypermetabolic site confirmed the metastatic lung tumor (**F**). A 79-year-old man with possible metastatic bone tumor was detected by CT(**G**), while the corresponding site showed no increased bone metabolism(**H**), the biopsy proved malignant. A 70-year-old man who represented with heterogenous density of the bone, the [^18^F]FDG PET/CT showed no abnormal lesions(**J**-**K**), pathological result showed ‘the tissue obtained was mainly bone marrow and fatty tissue and tumor cells were not seen(**L**)

All patients were required to fast for at least 6 h and undergo a peripheral blood sugar test to avoid hyperglycemia. Approximately 1 h after the intravenous injection of [^18^F]FDG [333-5^18^MBq (9-14mCi)], imaging was performed using an integrated PET/CT system (Discovery VCT; GE Medical Systems) from the head to the lower limbs with the following settings: CT scan, 120 V and 80 mA, 64 slices, with a slice thickness of 3.75 mm. PET scans were performed with 2.5 min per bed position. Finally, CT and PET images were reconstructed using ordered subset expectation maximization. Attenuation correction was performed using unenhanced CT. A senior nuclear medicine doctor evaluated all combined [^18^F]FDG PET/CT scans. The region of interest (ROI) around the bone lesions was drawn on [^18^F]FDG PET/CT images of each transaxial slice. SUVmax was defined as the peak value of the pixel with the highest count within the ROI. CT was performed regularly. The [^18^F]FDG PET/CT and CT images are shown in Fig. 1. All imaging data were anonymized and randomized. The CT and [^18^F]-FDG PET/CT examinations were read in consensus by three radiologists with 20 years of radiological experience.

#### Performance and costs

The performance of [^18^F]FDG PET/CT and CT was assessed in terms of the accuracy of detecting bone lesions, which were determined for detecting the malignant and benign lesions in the two groups by comparing the imaging test result with the reference standard.

If the imaging test showed an indicator of malignant metastases and the biopsy was unsatisfactory owing to the lack of tumor cells, patients underwent another invasive biopsy to exclude a possible malignant diagnosis. In the [^18^F]FDG PET/CT group, in which the imaging test itself could cover the entire body, patients underwent another biopsy via other hypermetabolic puncture sites. Due to the limited visual field of CT, patients in the CT group may need to undergo anothor CT test to detect possible metabolic concentration foci, and then may undergo a second biopsy via the puncture site determined by new CT results.

We consider a successful puncture as one in which we obtain the tissue for pathologic analysis after imaging localization. If the first puncture fails due to inaccurate imaging localization, a second puncture is required, which is usually successful. The cost of the second puncture was 8,266.34 RMB more than the cost of the first puncture.

We registered the diagnostic time as the time gap between the date of the first imaging test ([^18^F]FDG PET/CT or CT) and the date of the final accurate diagnosis. All intervals were calculated in calendar days including weekends and holidays. All data were obtained from the patients’ medical records.

#### Reference standard

All included patients underwent bone biopsy. A biopsy was performed by an interventional radiologist under CT guidance using the standard procedure of our radiology department. The puncture site was selected based on the presence of hypermetabolic bone lesions, represented by SUVmax, in the [^18^F]FDG PET/CT group. In the CT group, the puncture site was selected by an interventional radiologist based on the CT scan (Fig. 1). Pathologists first decalcified and evaluated bone specimens during routine work at our hospital. Only one tissue sample was obtained from each patient after a biopsy, and all biopsies yielded sufficient tissue to perform a pathological test. The reference standard was the pathological result of bone biopsy.

### Outcomes

The primary objective was to compare the accuracy, sensitivity, and specificity of [^18^F]FDG PET/CT and CT in diagnosing bone metastasis. The secondary outcome was the comparison of the second biopsy rate and cost-effectiveness. The experimental outcome was the application of metabolic indicators of [^18^F]FDG PET/CT and bone turnover markers to differentiate malignant metastatic bone lesions from benign lesions and to improve the diagnostic efficacy of [^18^F]FDG PET/CT.

### Statistical analysis

**Fig. 2:**
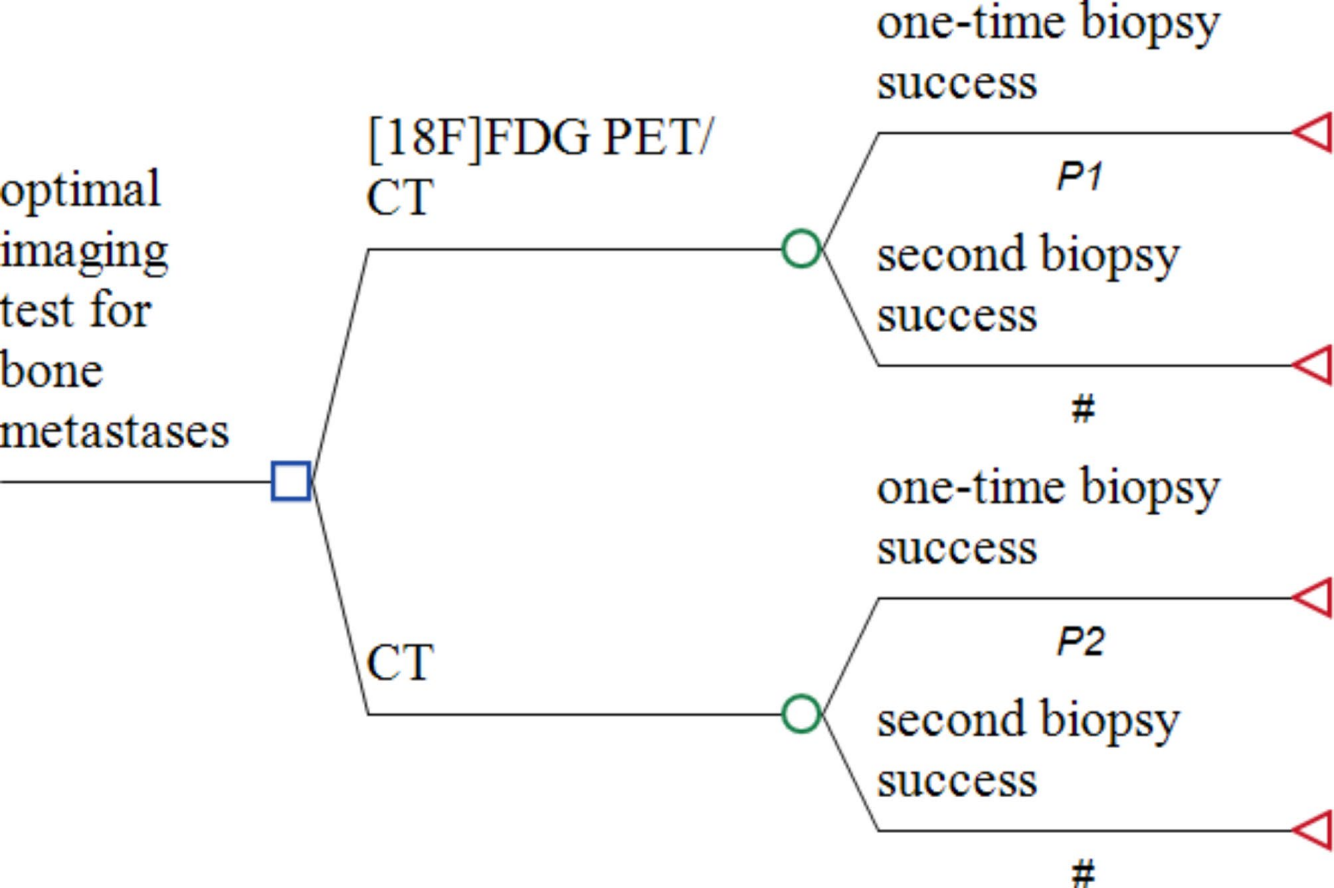
Decision tree illustrating the compared imaging test for bone metastases

The characteristics of the included patients were compared using the Fisher’s exact test for binary data and the Wilcoxon rank-sum test for non-normally distributed continuous data. All tests were two-sided, and P-values less than 0.05 were considered statistically significant. The statistical analyses were performed using STATA/IC version 15.1 (Stata Corp., LLC). Receiver Operating Characteristic (ROC) curves were drawn using MedCalc version 19.0.4 (MedCalc Software). The ROC curve was constructed to obtain the cutoff value of SUVmax and ALP in diagnosing bone metastases. Logistic regression analysis was performed to identify independent factors for the diagnosis of bone metastases. Variables with *P* < 0.05 in multivariate analysis were independent diagnostic factors. The area under the curve (AUC) was calculated separately, along with 95% confidence intervals (CI). The cutoff value was determined using the best Youden index on the ROC curves analyzed using MedCalc version 19.0.4. All diagnostic outcomes were based on patient-based analysis. The cost-effectiveness analysis was conducted using TreeAge Pro^®^ 2021, R2 software [14]. A simplifed model is shown in Fig. 2 and the full model diagram can be assessed in the supplementary.

## Results

### Baseline characteristics of patients

**Fig. 3:**
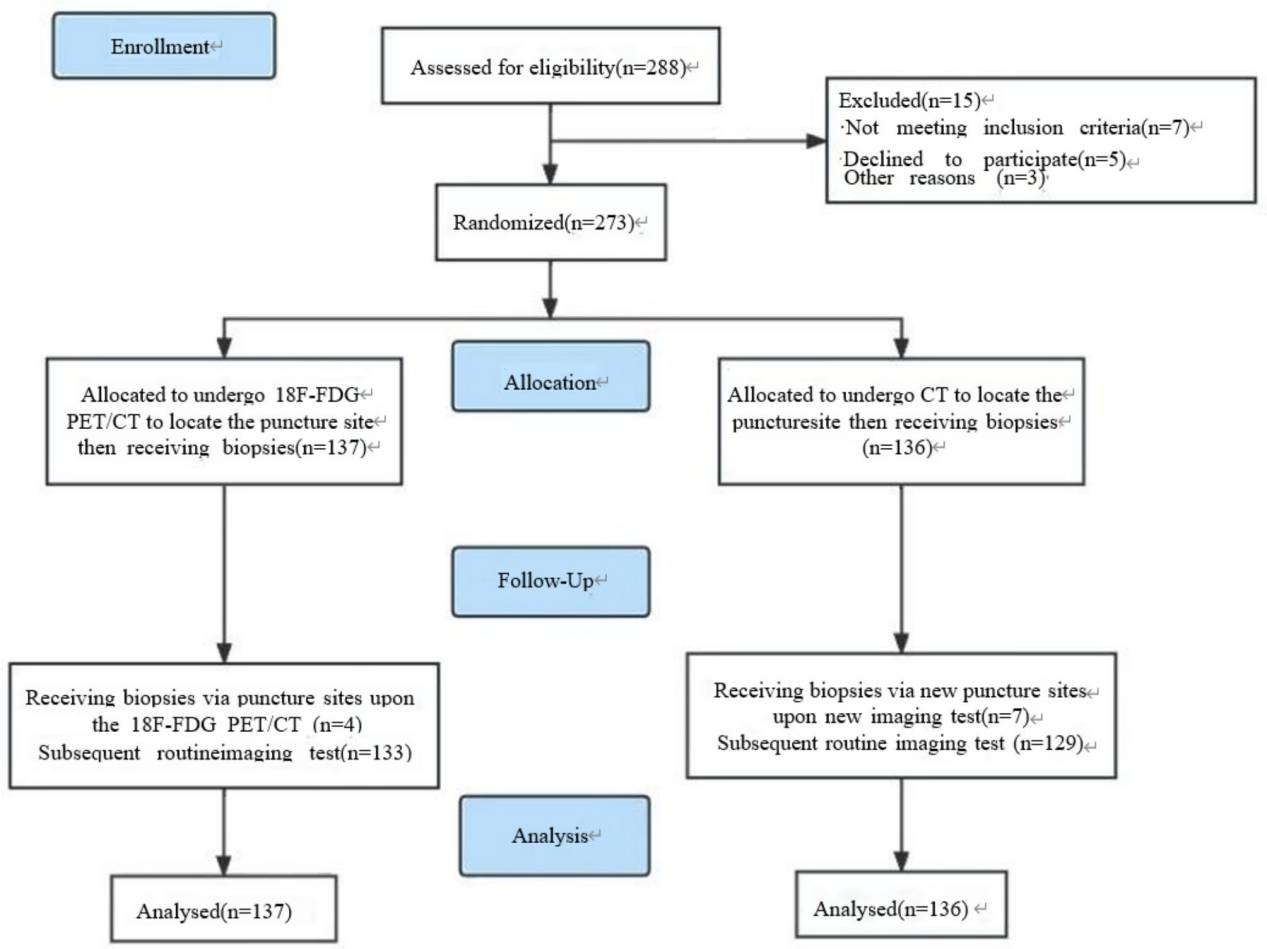
Flowchart of the prospective study

Between January 2020 and March 2021, 273 patients were enrolled in this prospective cohort study; 137 patients were randomly assigned to the [^18^F]FDG PET/CT group and 136 patients were randomly assigned to the CT group. All patients underwent bone biopsy at a site located by [^18^F]FDG PET/CT or CT (Fig. 3). The characteristics of the enrolled patients are summarized in Table 1. Sex, age, bone metastases character, and KPS score in the two groups have no statistical differences between the two groups (*P* > 0.05).

**Table 1:** The characteristic of enrolled patients

Patients Characteristics	[^18^F]-FDG PET-CT (*n* = 137)	CT (*n* = 136)	*P*-value
Gender			
Male, n (%)	90 (65.7)	75 (55.1)	0.075
Female, n (%)	47 (34.3)	61 (44.9)	
Age (years)			0.4226
Mean ± SD	57.11 ± 14.96	58.39 ± 11.11	
Bone metastases character, n (%)			0.0695
Lytic, n (%)	76 (33.6)	59 (43.4)	
Blastic, n (%)	24 (17.5)	23 (16.9)	
Mixed, n (%)	37 (48.9)	54 (39.7)	
KPS score			0.1019
≥ 80, n (%)	63 (46.0)	76 (55.9)	
60–70, n (%)	74 (54.0)	60 (44.1)	
Imaging test result			
Bone metastases, n (%)	83 (60.5)	89 (65.5)	0.4059
Benign bone lesions, n(%)	54 (39.5)	47 (34.5)	
Final diagnosis			0.2285
Bone metastases, n (%)	81 (59.1)	90 (66.2)	
Benign bone lesions, n(%)	56 (40.9)	46 (33.8)	

All bone biopsies were successfully performed. The final pathological findings for each group are presented in Supplementary Table 1. No significant differences in baseline were observed (*P* > 0.05).

### Diagnostic performance

81 patients had malignant bone metastases and 56 patients had benign bone lesions in [^18^F]FDG PET/CT group (Table S1). [^18^F]FDG PET/CT detected 80 out of 81 actual malignant metastases, and misinterpreted 3 benign lesions as malignant bone metastases. This resulted in a sensitivity of 98.8% (95%CI 93.3–99.9%) and a specificity of 94.6% (95%CI 85.1–98.9%).Of the 136 biopsies in the CT group, 89 were positive for malignant bone metastases and 47 were benign bone lesions. CT detected 83 out of 90 actual malignant metastases and misinterpreted 6 benign lesions as malignant bone metastases. This resulted in a sensitivity of 92.2% (95%CI 84.6–96.8%) and specificity of 86.9% (95%CI 73.7–95.1%)(Table 2).

**Table 2:** Comparison of the performance and cost between the [^18^F]FDG PET/CT and CT groups

	Imaging test		
	[^18^F]FDG PET/CT group	CT group	*P* valule
Diagnostic accuracy (n, %)	97.08 (133/137)	90.44 (123/136)	0.0232*
Diagnostic sensitivity (n, %)	98.76 (80/81)	92.22 (83/90)	0.0394*
Diagnostic specificity (n, %)	94.64 (53/56)	86.96 (40/46)	0.1134*
Second biospsy rate (n, %)	2.19 (3/137)	5.15 (7/136)	0.031*
Diagnostic time (mean ± SD, day)	18.33 ± 2.08	21.28 ± 1.25	0.021^#^

^*^χ^2^ test, ^#^ Independent-samples t-test

[^18^F]FDG PET/CT had a significantly higher sensitivity than CT for detecting malignant metastases (*P* = 0.0394), and the specificity of the two groups showed no difference (*P* = 0.1134). The accuracy of diagnosing bone lesions via [^18^F]FDG PET/CT was 97.1%, compared to 90.4% via CT, and [^18^F]FDG PET/CT was significantly superior to CT in terms of bone lesion diagnosis performance (*P* = 0.0232) (Table 2).

### Cost-effectiveness

**Fig. 4:**
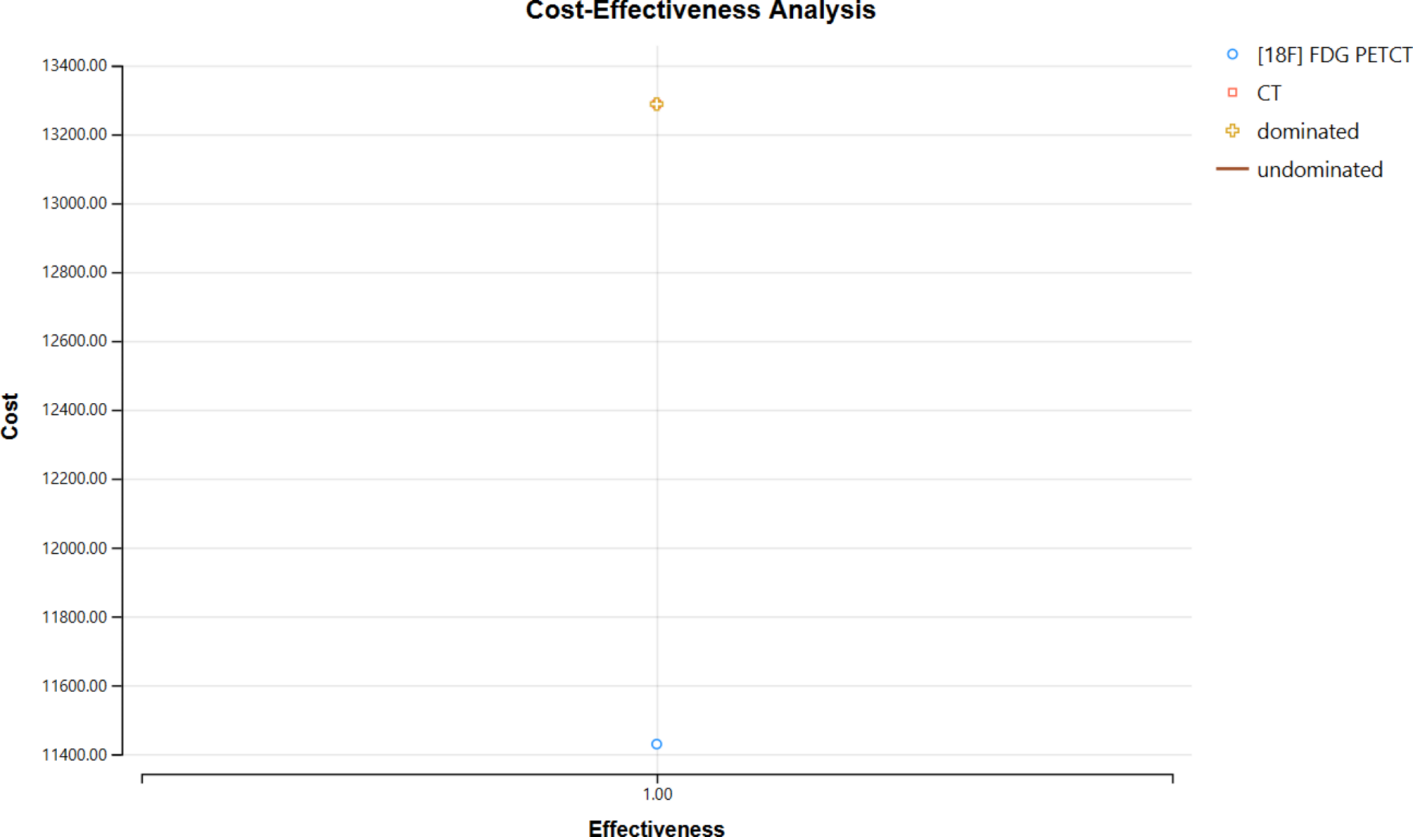
The cost-effectiveness analysis of [^18^F]FDG PET/CT and CT

In the [^18^F]FDG PET/CT group, the rate of a second biopsy resulting from an unsatisfactory biopsy was 2.19%, which was significantly lower than that of the CT group (5.15%; *P* = 0.031). The diagnostic time caused by the second biopsy was 18.33 ± 2.08 days in [^18^F]FDG PET/CT group, which significantly shorter than the 21.28 ± 1.25 days in CT group (*P* = 0.021)(Table 2). The average daily cost of treatment during the diagnostic time was 859.34 RMB per day. We compared the cost-effectiveness analysis of the two imaging modalities in terms of diagnostic time and secondary puncture, and the results of Treeage’s decision-tree modeling resulted in a superior cost-effectiveness of [^18^F]FDG PET/CT compared to CT (Table 3; Fig. 4).The cost of [^18^F] FDG PETCT is 11428.35 yuan, and the cost of CT is 13287.52 yuan; the incremental cost is 1859.17 yuan.

**Table 3:** Cost-effectiveness report of [^18^F] FDG PETCT and CT

Strategy	Dominance	Cost (rmb)	Incremental Cost (rmb)	Effectiveness
[^18^F] FDG PETCT	undominated	11428.35		1
CT	abs. dominated	13287.52	1859.166766	1

### Metabolic and blood test parameters combined to assist diagnosis

As summarized, age (median 63 vs. 54.5, *P* = 0.0308), ALP (median 126 vs. 87, *P* = 0.0011), and SUVmax (median 9.4 vs. 4.25, *P *< 0.001) were significantly higher in patients with malignant metastases than in those with benign lesions (Table S2). Univariate logistic regression analysis showed that age, ALP level, and SUVmax were significantly correlated with malignant lesions. Subsequent multivariate logistic regression analysis showed that ALP and SUVmax significantly correlated with bone metastases (*P* = 0.0011, *P *< 0.001)(Table 4).

**Table 4:** Patient characteristics of [^18^F]FDG PET/CT group and the univariate& multivariate logistic regression analysis results on bone metastases and benign lesions

Characteristics	Univariate Analysis OR (95% CI)	*P* value	Multivariate Analysis OR (95% CI)	*P* value
Age(year)	1.029 (1.005,1.054)	0.017	1.008 (0.974,1.044)	0.65
ALP (U/L)	1.038 (1.022,1.055)	< 0.001	1.035 (1.014,1.056)	0.001
Calcium (mmol/L)	1.833 (0.306,10.996)	0.507	0.653(0.062,6.851)	0.723
Phosphorus (mmol/L)	0.892 (0.340, 2.343)	0.817	1.546(0.409,5.833)	0.521
SUVmax	1.66(1.392,1.979)	< 0.001	1.515(1.263,1.818)	< 0.001

**Fig. 5:**
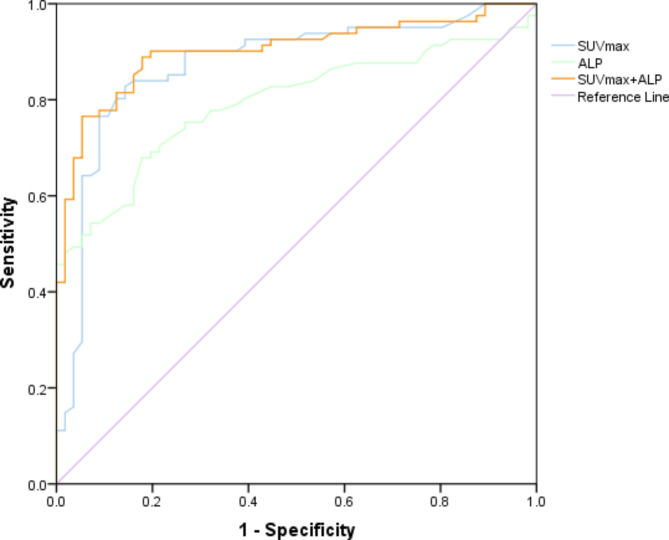
The ROC curve analysis of [^18^F]FDG PET/CT

ROC curve analysis showed that SUVmax > 6.3 could indicate malignant tumor metastases with an AUC of 0.873 (95% CI 0.806–0.924, *P *< 0.001), could indicate malignant tumor metastases, and ALP > 103 U/L also showed a tendency for tumor metastases with an AUC of 0.793 (95%CI 0.716–0.858, *P *< 0.001). With the two factors combined, when SUVmax of the lesions reached 6.3 and blood ALP are above 103 U/L, there was a possibility of 0.901 that the patient had a metastatic bone tumor (95%CI 0.839 to 0.946, *P *< 0.001) (Fig. 5; Table 5).

**Table 5:** Diagnostic characteristics of SUVmax, serum ALP and combined metrics

Diagnostic outcomes	SUVmax	ALP	Combined SUVmax + ALP
Cutoff value	6.3	103	
Sensitivity	82.72	67.90	76.54
Specificity	85.71	82.14	96.64
AUC	0.873	0.793	0.901
95% CI	0.806 to 0.924	0.716 to 0.858	0.839 to 0.946

[^18^F]FDG PET/CT can also be used to differentiate bone metabolic lesions due to bone metastases and inflammatory diseases. In [^18^F]FDG PET/CT group, we further subgrouped patients with bone metastases and inflammatory bone lesions. Univariate logistic regression analysis showed that age, ALP level, and SUVmax were significantly correlated with bone metastases. Subsequent multivariate logistic regression analysis showed that ALP levels and SUVmax significantly correlated with bone metastases (Table 6).

**Table 6:** Patient characteristics of [^18^F]FDG PET/CT group and the univariate& multivariate logistic regression analysis results on bone metastases and inflammatory bone lesions

Characteristics	Univariate Analysis OR (95% CI)	*P* value	Multivariate Analysis OR (95% CI)	*P* value
Age(year)	1.027(1.001,1.054)	0.042	1.007(0.972,1.044)	0.697
ALP (U/L)	1.025(1.011,1.040)	0.000434	1.025 (1.007.1.043)	0.007
Calcium (mmol/L)	1.999(0.271,14.722)	0.497	1.470(0.087,24.719)	0.789
Phosphorus (mmol/L)	0.747(0.256,2.180)	0.593	1.657(0.345,7.959)	0.528
SUVmax	1.641(1.361,1.979)	0.00000021519	1.600991(1.320,1.941)	0.000002

**Fig. 6:**
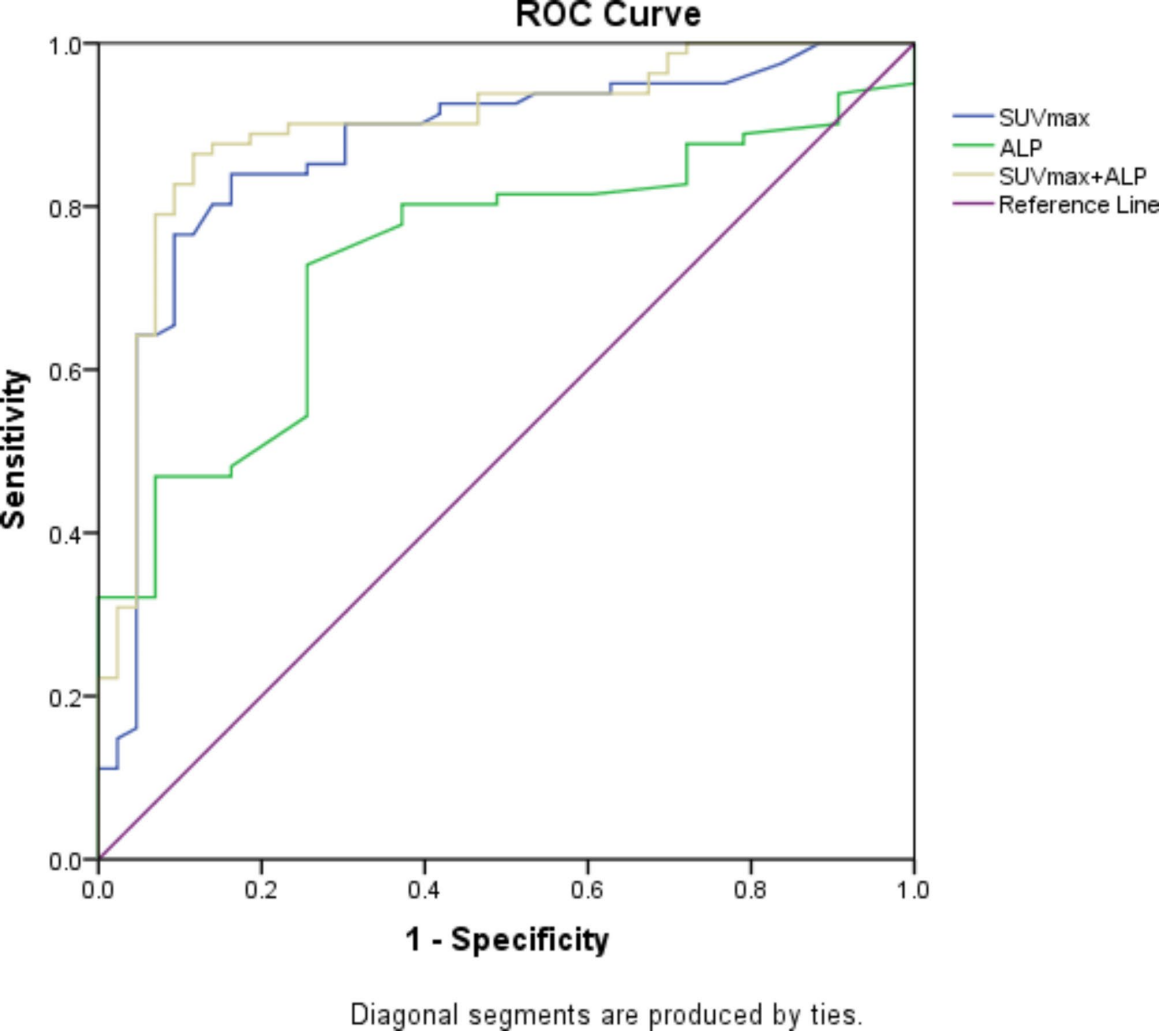
The ROC curve analysis of subgroup in [^18^F]FDG PET/CT group

ROC curve analysis showed that SUVmax > 6.2 could indicate bone metastases with an AUC of 0.871 (95% CI 0.799 to 0.924, *P *< 0.001), and ALP > 87 U/L also showed a tendency for bone metastases with an AUC of 0.738 (95%CI 0.651 to 0.813, *P *< 0.001). With the two factors combined, there was a possibility of 0.898 that the patient had bone metastases (95%CI 0.837 to 0.958, *P *< 0.001) (Fig. 6; Table 7).

**Table 7:** Diagnostic characteristics of SUVmax, serum ALP and combined metrics on bone metastases and inflammatory bone lesions

Diagnostic outcomes	SUVmax	ALP	Combined SUVmax + ALP
Cutoff value	6.2	87	
Sensitivity	83.95	72.84	76.54
Specificity	83.72	74.42	96.64
AUC	0.871	0.738	0.898
95% CI	0.799 to 0.924	0.651 to 0.813	0.837 to 0.958

## Discussion

Bone metastasis is an indicator of advanced lesions, and timely diagnosis is very important. Bone metastases, which are immeasurable lesions, can occur in the entire body of patients with tumors [15]. Bone biopsy is the gold standard for diagnosing bone metastases.The most widely used techniques to determine bone biopsy site are [^18^F]FDG PET/CT and CT [8]. Our study was a prospective study based on a medical center specializing in various bone diseases that examined patients with possible bone metastases using [^18^F]FDG PET/CT and CT, and then performed percutaneous biopsy under CT guidance. The main finding of our study is that [^18^F]FDG PET/CT is more suitable than CT in determining the bone biopsy site for suspect bone metastases.

CT-guided biopsy is often recommended for the initial diagnosis of skeletal lesions and has been accepted as safe, minimally invasive, and cost-effective for diagnostic confirmation, thereby preventing the need for riskier and more invasive open surgical biopsy procedures in most patients. Previous studies have also described the success rate of CT-guided biopsy in identifying morphologically clear bone lesions to be in the range of 69–90%; however, the success rate of biopsies may be unexpectedly lower for lesions that are characterized by their metabolic information rather than by anatomic structure [7, 16]. The success rate of CT-guided biopsy is unsatisfactory. [^18^F]FDG PET/CT can not only target morphologically clear lesions but also metabolically active areas. [^18^F]FDG PET/CT inherent quantitative nature enables accurate, reproducible measurements of radiopharmaceutical uptake in the tumour during diagnostic work-up [17]. [^18^F]FDG PET/CT is also recommended as a workup for potential bone metastases (evidence-level category 2 B) by the National Comprehensive Cancer Network (NCCN) guidelines version 3.2023.

Many studies have compared the detection efficiencies of these two imaging tests, but none of them have been prospective, our study was the first prospective study. In a retrospective study by Guo et al., [^18^F]FDG PET/CT yielded a high diagnostic success rate for evaluating bone lesions in patients [9]. The first-time diagnostic success rate of biopsy was 96.1%, which was inferior to the success rate of the [^18^F]FDG PET/CT group in our study. A retrospective study by Wu et al. reported that the overall diagnostic yield of [^18^F]FDG PET/CT in initial biopsies was significantly higher than that in the CT group [15]. In a study by Cornelis et al., [^18^F]FDG PET/CT allowed high diagnostic success of percutaneous biopsies for metabolically active lesions that are difficult to see with conventional cross-sectional imaging [18]. Other retrospective analyses have shown consistent results, the superiority of [^18^F]FDG PET/CT in retrospective studies is obvious [19]. Our study compared [^18^F]FDG PET/CT with CT in a prospective randomized controlled trial for the first time and confirmed that [^18^F]FDG PET/CT has higher accuracy and sensitivity than CT. Moreover, [^18^F]FDG PET/CT ‘s role extends beyond initial diagnosis, as it can also be used to monitor the response to treatment, assess the progression of disease, and detect recurrence. The sensitivity and specificity of [^18^F]FDG PET/CT in detecting metabolic changes make it a cornerstone in the multidisciplinary approach to cancer care, particularly for those cancers known to have a high propensity for bone metastasis, such as breast, prostate, and lung cancers [20, 21].

[^18^F]FDG PET/CT is more advantageous than CT in terms of cost-effectiveness analysis for identifying sites for bone biopsy. Considering the high cost of [^18^F]FDG PET/CT, CT may be more practical in this field. However, CT can lead to false-negative diagnoses and unsatisfactory pathological results that could not reach diagnoses [22]. In our study, seven patients in CT group did not get diagnoses during the first pathological biopsy, compared to [^18^F]FDG PET/CT, only three patients failed to reach diagnoses at the first biopsy. Owing to the limitations of CT devices, if the first biopsy didn’t get the appropriate tissue, a secondary puncture must be done. The diagnostic time was prolonged due to the second biopsy and the costs went up. Many other studies have shown that [^18^F]FDG PET/CT could significantly reduce the cost of cancer management by improving the accuracy of both diagnosis and staging, thereby helping to avoid expensive, futile intervetions and associated side effects [23–26].Overall, [^18^F]FDG PET/CT has the potential to improve the diagnoses and reduce the cost burden over time to the healthcare system. However, the potential of [^18^F]FDG PET/CT as a tool to help in the management of cancer patients has not yet been reflected in the extent of its adoption.

In our study, we identified that SUVmax can be utilized to assist in determining the benign or malignant nature of suspicious bone lesions. High uptake of SUVs represents an area with active metabolism. Metabolically active bone tissue may be undergoing the process of bone turnover, which may be some inflammatory diseases or tumor bone metastases. As a metabolic index, SUV can help distinguish benign from malignant masses, or biologically aggressive from non-aggressive regions of malignant masses [16]. Yao et al. reported an SUVmax of 5 to predict bone metastasis, and that SUVmax could be a valuable noninvasive predictor of EGFR mutations in lung adenocarcinoma [27, 28].Our study showed that SUVmax > 6.3 indicates that the bone lesion is malignant, which is higher than that reported in the literature, and can be attributed to a minority of extreme values [29]. Compared with previous studies, our study had the highest AUC [27, 29]. These cutoff values could be helpful when choosing the most suitable site for bone biopsy.

We found that ALP, a bone turnover marker (BTM), can be used to assist in determining the nature of bone lesions. BTMs are substances released in the blood and urine that can reflect bone resorption and formation during the remodeling process and can be used to predict the risk of bone metastases, including ALP, calcium, and phosphorus [12, 30]. Univariate and multivariate logistic regression analyses were performed to determine that ALP were associated with malignant bone metastases. Serum calcium and phosphorus levels are strongly affected by disease and diet; therefore, they are not stable indicators that can be used to diagnose bone metastases. In contrast, ALP indicates relatively stable bone metabolism.

This study has some limitations. The prospective study is single-center and the sample size was limited. SUVmax exhibits low reproducibility, and its values can be significantly influenced by variations in scanner protocols. We would conduct a large multi-center clinical trial to validate our findings, with good quality control in terms of equipment and personnel to ensure as much reproducibility and consistency as possible in our findings. There are many other BTMs such as bone steocalcin (OC), osteoprotegerin (OPG) and type 1 collagen protein C-terminal cross-linking peptide (CTX) that can be used as indicators for diagnosing bone metastasis[31]. In this article we only include ALP, calcium, and phosphorus. More indicators can be used to assist in diagnosis.

## Conclusion

[^18^F]FDG PET/CT has better performance and cost-effectiveness than CT for localizing the bone biopsy site for suspect bone metastases. Thus, we recommend [^18^F]FDG PET/CT as an imaging test to localize the site of bone biopsy.

## Electronic supplementary material

Below is the link to the electronic supplementary material.

Supplementary Material 1

## Data Availability

No datasets were generated or analysed during the current study.

## Abbreviations

CT: Computed tomography
[^18^F]FDG PET/CT: [^18^F] Fluorodeoxyglucose positron emission tomography/computed tomography
AUC: Area under ROC curve
ALP: Alkaline phosphatase
SREs: Skeletal-related events
QoL: Quality of life
SUV: Standardized uptake values
ROI: Region of interest
ROC: Receiver operating characteristic
CI: Confidence intervals
NCCN: National Comprehensive Cancer Network
BTM: Bone turnover markers
OC: Osteocalcin
P1NP: Type 1 procollagen N-terminal peptide
OPG: Osteoprotegerin
NTX: Type 1 collagen protein N-terminal crosslinking peptide
CTX: Type 1 collagen protein C-terminal cross-linking peptide
