# Correction: [^18^F]FDG PET/CT performs better than CT in determining the bone biopsy site : randomized controlled clinical trial

**DOI:** 10.1186/s40644-026-01092-y

**Published:** 2026-08-06

**Authors:** Yujie Chang, Yifeng Gu, Shunyi Ruan, Shengyuan Xu, Jing Sun, Zhiyuan Jiang, Guangyu Yao, Zhiyu Wang, Hui Zhao

**Affiliations:** 1 Internal Oncology, Shanghai Jiao Tong University Affiliated Sixth People’s Hospital, Shanghai, 200233 China; 2 Interventional Radiology, Shanghai Jiao Tong University Affiliated Sixth People’s Hospital, Shanghai, China; 3 Mailman School of Public Health, Columbia University, New York, USA

**Cancer Imaging (2024) 24:160**

10.1186/s40644-024-00804-6

Following publication of the original article [1], we were notified that the 4th author’s name was incorrectly listed as “Shengyu Xu” instead of “Shengyuan Xu.” The error was due to an incorrect romanized pinyin spelling of the author’s Chinese name.

The original article has been corrected.
