# Supplementary material for: [18F]FDG PET/CT performs better than CT in determining the bone biopsy site : randomized controlled clinical trial
Source: Cancer Imaging. 2024 Nov 24;24:160. doi: 10.1186/s40644-024-00804-6 (PMC11587546; doi:10.1186/s40644-024-00804-6)
Supplement: Supplementary file 1 — Supplementary Material 1 [file 40644_2024_804_MOESM1_ESM.docx]

Supplementary

S1 Comparison of the final diagnoses between the 18F-FDG PET/CT and CT groups

| Bone biopsy final diagnosis | PET-CT (n=137) | CT (n=136) | *P* value |
| --- | --- | --- | --- |
| Bone metastases | 81 | 90 | 0.4824 |
| Lung cancer metastases | 60 | 69 |  |
| Gastrointestinal cancer metastases | 2 | 4 |  |
| Urinary system cancer metastases | 11 | 7 |  |
| Breast cancer metastases | 4 | 2 |  |
| Thyroid cancer metastases | 1 | 4 |  |
| Reproductive system metastases | 3 | 4 |  |
| Benign bone diseases | 56 | 46 | 0.0717 |
| Bone trabecula/cartilage tissue | 9 | 11 |  |
| Bone inflammation | 37 | 18 |  |
| Degenerative diseases | 4 | 8 |  |
| Osteomyelitis | 4 | 4 |  |
| Tuberculosis | 2 | 5 |  |

S2 Patient characteristics of 18F-FDG PET/CT group

| Characteristics | benign lesions  (n=56) | malignant metastases  (n=81) | *P* value |
| --- | --- | --- | --- |
| Gender |  |  | 0.0564 |
| Male, n (%) | 42 | 48 |  |
| Female, n (%) | 14 | 33 |  |
| Age (years) |  |  | 0.0150 |
| Mean | 53.393 | 59.679 |  |
| Median | 54.5 | 63.0 |  |
| Bone metastases character, n (%) |  |  | 0.9220 |
| Lytic, n (%) | 32 | 44 |  |
| Blastic, n (%) | 9 | 15 |  |
| Mixed, n (%) | 15 | 22 |  |
| KPS score |  |  | 0.0672 |
| ≥80, n (%) | 31 | 32 |  |
| 60~ 70, n (%) | 25 | 49 |  |
| ALP(U/L) |  |  | 0.0001 |
| mean | 88.857 | 170.86 |  |
| median ALP | 87 | 126 |  |
| Calcium(mmol/L) |  |  | 0.5280 |
| mean | 2.238 | 2.260 |  |
| median calcium | 2.290 | 2.230 |  |
| Phosphorus(mmol/L) |  |  |  |
| mean | 1.328 | 1.314 | 0.8186 |
| median phosphorus | 1.33 | 1.29 |  |
| SUVmax |  |  |  |
| mean SUVmax | 4.759 | 10.07 | ＜0.001 |
| Median SUVmax | 4.25 | 9.4 |  |


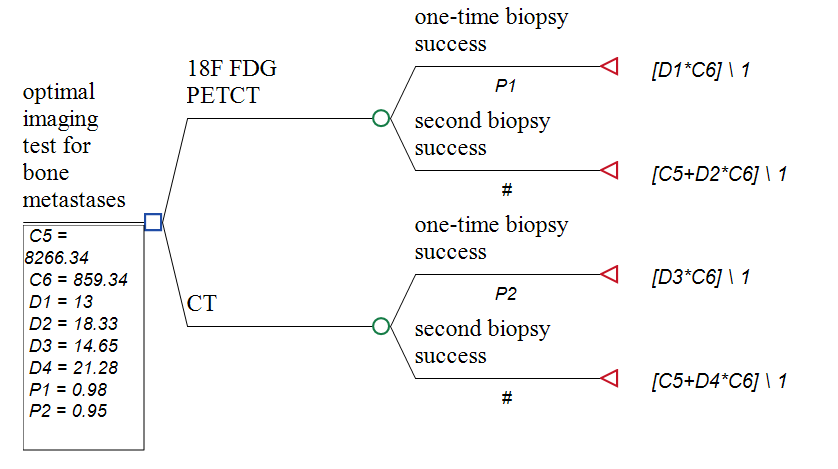


Figure S3. The full model diagram of decision tree illustrating the compared imaging test for bone metastases.
